# Metformin and Diabetic Kidney Disease: A Mini-Review on Recent Findings

**Published:** 2014-09-12

**Authors:** Hamid Nasri, Mahmoud Rafieian-Kopaei

**Affiliations:** 1 Department of Nephrology, Division of Nephropathology, Isfahan University of Medical Sciences, Isfahan; 2 Medical Plants Research Center, Shahrekord University of Medical Sciences, Shahrekord, Iran

**Keywords:** Metformin, Kidney Disease, Diabetes, Anti-diabetic Agents

## Abstract

Metformin, an oral anti-diabetic agent in the biguanide class is a widely prescribed drug to treat high blood glucose in patients with type 2 diabetes mellitus. Metformin has three different roles, including blood glucose regulatory effect, protection of kidney tubular cell by acting as an effective antioxidant and finally ameliorative effect on diabetic kidney disease through saving the podocytes. Therefore, diabetic patients may benefit from all of these three distinct ameliorative effects.

## Introduction

Diabetes mellitus is now the major cause of end-stage renal failure around the world in both developed and underdeveloped countries^[1,2]^. It is the primary diagnosis causing renal failure in 20–40% of people starting treatment for end-stage kidney failure worldwide^[2^^-^^4]^. Vascular and glomerular damages have been found the main features of diabetic nephropathy, however tubular atrophy also plays a major role in the disease^[3,4]^.

***Mechanisms of Diabetic Kidney Disease***

Apoptosis contributes to the development of diabetic kidney disease^[5,6]^. It is possible that high glucose enhances apoptosis, a form of programmed cell death displayed by cell shrinkage, DNA fragmentation or chromatin condensation in a variety of cell types, especially renal proximal tubular epithelial cells^[7,8]^. Moreover, diabetic kidneys are mainly prone to acute tubular injury in various clinical situations, such as dehydration or post renal obstruction^[8-10]^. It is also well determined that hyperglycemia by itself is an independent risk factor for acute tubular injury under these conditions. High blood glucose activates the generation of free radicals and oxidative stress in tubular cells. Reactive oxygen species (ROS) are thought to be important mediators for several biologic responses including proliferation and apoptosis^[8-13]^. Indeed, besides apoptosis of proximal tubular epithelial by hyperglycemia, other cell types including podocytes will also be affected by hyperglycemia^[10-13]^. Thus one of beneficial treatments in the diabetic kidney disease is using medication for inhibiting or reducing apoptosis to save renal tubular cells and podocytes. In a study on patients with type 2 diabetes mellitus (T2DM), inhibition of the rennin-angiotensin–aldosterone system using an angiotensin converting enzyme (ACE) inhibitor or blocking the effect of angiotensin II through using an angiotensin receptor blocker decreased the progression from normoalbuminuria to microalbuminuria^[12-19]^ and slowed the development of end-stage renal disease (ESRD)^[15-20]^. Thus, the use of an ACE inhibitor or angiotensin receptor blocker (ARB) or combination of both is now standard therapy for patients with diabetic nephropathy along with glucose, lipid and blood pressure control^[2-9]^. 

***Renoprotective Effects of Metformin***

Metformin, an oral anti-diabetic agent in the biguanide class is a widely prescribed drug to treat high blood glucose in patients with T2DM^[21,22]^. Recent studies have recognized that metformin possesses antioxidant effects, too. Reduction of apoptosis, in endothelial cells as well as inhibition of vascular endothelial cell dysfunction was also found during metformin treatment^[23^^-^^25]^. The beneficial action of this drug is achieved through activation of adenosine monophosphate-activated protein kinase (AMPK). This enzyme plays an important role in protecting cellular functions under energy-restricted conditions. Many evidences, confirm that AMPK activation by metformin is secondary to its effect on the mitochondria as the primary target of this drug. There is evidence that the beneficial effect of metformin might be due to its mild inhibition of the mitochondrial respiratory chain, while the serious role of mitochondria in cell death is of significance, and protecting the mitochondria has become a pro-survival cell strategy ^[16-25]^. It is possible that the role of mitochondria in programmed cell death is associated with the release of apoptotic signaling molecules. ROS production by mitochondria may also lead significantly to cell injury. Some years ago, Morales et al found that gentamicin-induced renal tubular injury is abolished by metformin^[26]^. It is clear that, reactive oxygen species play a key role in the toxicity of gentamicin, resulting in acute kidney injury, and gentamicin is a mitochondrial toxin that can imply its toxic effects when excreted by the kidney. Mitochondrial toxicity can also be mediated by ROS as mentioned above^[13,19,20,22-25]^. ROS is normally produced at low levels by mitochondria, conversely under pathological situations the intracellular and intra mitochondrial ROS content may be increased. When in certain conditions, intracellular ROS content reaches a toxic level, results in oxidative damage to the mitochondria causing cell death and malfunctioning of the organ^[13,19, 20,22-25]^. To test the potential renoprotective effects of metformin against gentamicin-induced renal damage and also finding out whether postponed treatment with metformin in acute kidney injury exerts similar benefits on gentamicin renal toxicity, we conducted a study on rats. In this study, metformin protected and also improved gentamicin- induced acute renal injury, hence, this drug might be effective for protection of tubular cells^[27]^. More recently, we also investigated the efficiency of co-administration of garlic juice and metformin for protection against gentamicin-kidney tubular injury in 70 rats. The results of this investigation showed that metformin and garlic extract or their combination have both curative and protective property against gentamicin renal-toxicity^[28]^. Accordingly, Li and colleagues showed that co-administration of metformin and gentamicin for 13 days efficiently reversed gentamicin-induced kidney injury^[29]^. Thus, these findings provide further evidence for the attribution of metformin in its kidney protective efficacy besides its well-known blood glucose regulatory action^[22-28]^. 

 While, diabetic kidney disease is one of the most important complications of this illness^[29-36]^ and metformin is widely used in this patients, especially in T2DM, recently much attention has also been directed on the possible protective and ameliorative properties of metformin in diabetic renal disease. Hyperglycemia intensifies oxidative stress and generation of ROS, which have a critical role in the pathogenesis of diabetic renal disease^[23-30]^. Indeed, metformin treatment caused significant restoration in diabetic renal disease-induced oxidative stress mRNA levels^[23-27]^. Various evidences suggest that ROS overproduction may be the key starting event that results in long-term development of problems of diabetes. ROS generation by oxidative stress causes cell death^[20-27]^. As it was mentioned previously, apoptosis is implicated in the pathogenesis of diabetic nephropathy and ROS is an inducer of apoptosis in various cell types containing podocytes^[22-27]^. Interestingly Kim et al conducted a study using metformin in diabetic rats. They observed the repair of podocytes by metformin treatment in diabetic rats. They suggested that diabetes-induced podocyte loss in diabetic nephropathy could be diminished by metformin^[37-40]^. They also found that the density of podocytes decreases in diabetic rats along with increased albumin excretion. Podocyte apoptosis has been identified to associate with increasing albuminuria. Moreover, there are evidences for the role of intracellular ROS as potent inducers of podocyte apoptosis. Kim et al also observed that the phosphorylation of AMPK was decreased in the kidney of diabetic rats, and metformin could reestablish its alteration. Therefore, metformin might exert some of its effects by amendment of renal oxidative stress^[34]^. Thus one might suggest metformin to inhibit the advanced glycation end-products and improve the free-radical defense system, hence, preventing the diabetic renal injury^[3]^. 

 It is well understood that the injury to the podocytes leads to occurrence of proteinuria^[32-36]^. Therefore the loss of glomerular podocytes precedes and predicts the onset of diabetic nephropathy and may be an early pathological manifestation of diabetic kidney disease. Metformin significantly decreased albuminuria in patients with T2D^[34,39,40]^. Previous studies have also shown the beneficial effects of metformin on reduction of macrovascular morbidity and mortality, suggesting anti-atherogenic, antioxidant and anti-inflammatory effects^[21-25]^. Furthermore, metformin significantly decreased albuminuria in patients with T2DM^[40-43]^. 

## Conclusion

Therefore, it is reasonable to interpret that metformin has three different roles, including: blood glucose regulatory effect, protection of kidney tubular cell by acting as an effective antioxidant and finally ameliorative effect on diabetic kidney disease through saving the podocytes. Therefore, diabetic patients may benefit from all of these three distinct ameliorative effects. However, it should be noted that, according to the contraindication of metformin in the estimated glomerular filtration rates (eGFR) of less than 30 mL/minute, we cannot suggest this drug for renal tubular cell protection in human studies. Hence, we suggest to more investigation on this subject.
